# MetaboLINK is a novel algorithm for unveiling cell-specific metabolic pathways in longitudinal datasets

**DOI:** 10.3389/fnins.2024.1520982

**Published:** 2025-01-13

**Authors:** Jared Lichtarge, Gerarda Cappuccio, Soumya Pati, Alfred Kwabena Dei-Ampeh, Senghong Sing, LiHua Ma, Zhandong Liu, Mirjana Maletic-Savatic

**Affiliations:** ^1^Jan and Dan Duncan Neurological Research Institute at Texas Children’s Hospital, Houston, TX, United States; ^2^Department of Pediatrics-Neurology, Baylor College of Medicine, Houston, TX, United States; ^3^College of Natural Sciences and Mathematics, University of Houston, Houston, TX, United States; ^4^Shared Equipment Authority, Rice University, Houston, TX, United States; ^5^Department of Neuroscience, Baylor College of Medicine, Houston, TX, United States

**Keywords:** metabolome, PCA, Glasso, MetaboLINK, hESC, embryonic bodies, rosettes, neuroprogenitors

## Abstract

**Introduction:**

In the rapidly advancing field of ‘omics research, there is an increasing demand for sophisticated bioinformatic tools to enable efficient and consistent data analysis. As biological datasets, particularly metabolomics, become larger and more complex, innovative strategies are essential for deciphering the intricate molecular and cellular networks.

**Methods:**

We introduce a pioneering analytical approach that combines Principal Component Analysis (PCA) with Graphical Lasso (GLASSO). This method is designed to reduce the dimensionality of large datasets while preserving significant variance. For the first time, we applied the PCA-GLASSO algorithm (i.e., MetaboLINK) to metabolomics data derived from Nuclear Magnetic Resonance (NMR) spectroscopy performed on neural cells at various developmental stages, from human embryonic stem cells to neurons.

**Results:**

The MetaboLINK analysis of longitudinal metabolomics data has revealed distinct pathways related to amino acids, lipids, and energy metabolism, uniquely associated with specific cell progenies. These findings suggest that different metabolic pathways play a critical role at different stages of cellular development, each contributing to diverse cellular functions.

**Discussion:**

Our study demonstrates the efficacy of the MetaboLINK approach in analyzing NMR-based longitudinal metabolomic datasets, highlighting key metabolic shifts during cellular transitions. We share the methodology and the code to advance general ‘omics research, providing a powerful tool for dissecting large datasets in neurobiology and other fields.

## 1 Introduction

Recent advancements in high-throughput technologies have significantly expanded the volume of biological datasets, transforming the landscape of network analysis in ‘omics research. As the omics field grows, there is an urgent need for robust and statistically reliable models to decode the complexities of big data. This need is especially critical in the rapidly evolving field of metabolomics, where comprehensive and precise analytical tools are essential for uncovering intricate metabolic networks.

Metabolomics, employing mass spectrometry (MS) or nuclear magnetic resonance spectroscopy (NMR), has emerged as a high-throughput tool crucial for obtaining a comprehensive perspective on cellular metabolic function both *in vitro* and *in vivo* (Maletic-Savatic et al., 2008; Tang et al., 2018; Arnold et al., 2015; Choi et al., 2018; Vingara et al., 2013; Bonomo et al., 2020). The rich and complex data often result in high-dimensional datasets that pose significant challenges in analysis and interpretation, especially in longitudinal studies. Traditional statistical methods frequently struggle with these complexities, making it challenging to identify, reproduce, and validate biomarkers or to elucidate relevant metabolic pathways. These challenges are particularly pronounced in longitudinal studies, where the need to track changes over time adds an additional layer of complexity. Recent advancements in analytical algorithms have addressed some of these challenges. For instance, advanced dimensionality reduction techniques like T-Distributed Stochastic Neighbor Embedding (TSNE) and Uniform Manifold Approximation and Projection (UMAP) have enhanced the visualization and interpretation of complex data (Yang et al., 2021). However, while useful, these methods still require careful application to avoid oversimplification and loss of critical information (Narayan et al., 2021; Zhai et al., 2022). For example, the inter-cluster distances and the long-range distance are difficult to interpret because of the adoption of the conditional probability by these methods. Two clusters close under UMAP or TSNE coordinates maybe be far apart in the original dimensions, which introduces challenges for populations with transitional functional states. Furthermore, the variance of clusters in UMAP and TSNE does not correlate with the actual variance. Instead, it is largely driven by the sample size and local density of data points. Consequently, interpreting the resulting UMAP becomes challenging, as it remains unclear whether the observed patterns represent a biological process of interest, a confounding biological factor, or a technical artifact.

Here, we introduce an innovative approach for longitudinal metabolomics data analysis, addressing a standardized and unbiased dimensionality reduction, variable selection, and data integration. Our method seamlessly combines principal component analysis (PCA) loadings with the graphical lasso (GLASSO) to reveal subnetworks in metabolomics datasets (Allen and Maletić-Savatić, 2011; Allen et al., 2013; Peterson et al., 2013; Gewers et al., 2022; Friedman et al., 2010). We validated the effectiveness of PCA-GLASSO (referred to as MetaboLINK from now on) for metabolomics analysis of human embryonic stem cell (hESC)-derived neural cell populations. We chose to focus on neural cells because of the critical role metabolism plays in proliferation and differentiation of these cells (Fawal and Davy, 2018; Erceg et al., 2008; Heiden et al., 2009; Iwata et al., 2023). Also, dysregulation of metabolic pathways has been implicated in various neurodevelopmental disorders, autism spectrum disorder, attention deficit hyperactivity disorder, and intellectual disabilities (Khaliulin et al., 2024). Furthermore, neurological symptoms (intellectual disability, epilepsy) occur in more than 50% of patients affected by inborn errors of metabolism (Žigman et al., 2021), supporting the concept that metabolic impairment can derail neural cell functioning. Understanding how metabolic changes influence neural metabolism is vital for unraveling the underlying mechanisms of these conditions and for identifying potential biomarkers and therapeutic targets (Urbán and Guillemot, 2014).

Designed for clear interpretation, MetaboLINK simplifies the exploration and pattern recognition of complex data. By applying MetaboLINK, we have identified cell type biomarkers across various neural developmental stages. Beyond neuro-metabolomics, MetaboLINK holds potential for analyzing large-scale, high-dimensional datasets in diverse fields and applications, including cancer research, pharmacometabolomics, cardiovascular disease, genetics, and environmental exposure studies. Its versatility makes MetaboLINK a significant advancement, by offering unique capabilities for revealing dynamic network patterns over time.

## 2 Methods

### 2.1 Neural fate commitment of human embryonic stem cells (hESCs)

To decipher a catalogue of metabolic biomarkers pertinent to glutamatergic neuronal commitment, without the interference of complex genetic background diversity, we used hESC (H9) line. hESCs were maintained in an established feeder-free culture system *in vitro* prior to neuro-ectodermal lineage commitment (Szczerbinska et al., 2019). The stage-specific differentiation to glutamatergic neurons is comprised of four distinct stages “*hESCs-to-Embryonic Bodies (EBs)-to-Rosettes-to-human Neuroprogenitors (hNPCs)-to-Neuron.”* These stages were established *in vitro* using modified protocols (Cho et al., 2008; Boisvert et al., 2013). To attain these stages, we prepared four different combinations of media for sequential progression of neural commitment: namely, neural induction medium (NIM), neural proliferation media 1 and 2 (NPM1, NPM2), and terminal differentiation media (TDM). We used NIM to induce neuro-ectodermal lineage specification, marked by formation of EBs on 4^th^ day *in vitro* (DIV). During induction and the early stage of neural rosette formation, we used both BMP/TGF inhibitors. To block non-neuronal lineages, we used Dorsomorphin (4 μM) + SB431542 (10 μM) combination. Following induction, we used NPM1 with bFGF/EGF (20 ng/mL) and Cyclopamine (1 μM) to induce proliferation of neural rosettes. Rosettes were dissociated into a single cell suspension with Accutase (Gibco) and seeded directly onto freshly prepared poly-L-ornithine (PLO, 10 mg/mL) and Laminin (LN, 1 mg/mL)-coated culture plates in NPM1 to develop hNPCs. Dorsal patterning of hNPCs was continued using NPM2 with a specialized cocktail of growth factors IGF+ BDNF+ GDNF (all 10 ng/mL) along with Cyclopamine (1 μM) for inhibiting ventral patterning into GABAergic neurons. Committed dorsally patterned hNPCs were seeded onto new PLO/LN coated culture dishes (100 mg/mL and 10 mg/mL respectively) and were driven to maturation using TDM with IGF, BDNF, and GDNF (all 10 ng/mL), and cAMP (100 μM). To prevent clumping of mature neurons in culture, we used 2 μg of LN in the media.

### 2.2 Flow cytometry of hESC-derived neural cell populations

To characterize stage-specific populations, we employed flow cytometry (BD Bioscience) (Yuan et al., 2011). We used cell specific markers to examine cell population purity at each stage: CD24 for EBs, ZO-1 for Rosettes, Nestin for hNPCs, and MAP2 and vGLUT1 for neurons. We used two markers for neurons to establish the glutamatergic neuron population in our cultures. Flow cytometry data were analyzed using FlowJo software.

### 2.3 Immunocytochemistry of hESC-derived neural cell populations

We validated hESCs and their guided neural progeny *in vitro* using primary antibodies against stage-specific markers such as CD184 (Anti-CD184 Mouse, Invitrogen) and CD24 (Anti-CD24 Rat, Invitrogen) for EBs; Pax6 (Anti-Pax6, Avain, Santa Cruz) and ZO-1 (Anti-ZO-1 Rabbit, Invitrogen) for Rosettes; Nestin (Millipore) for hNPCs; VGLUT1(Anti-VGlut1 rabbit, Invitrogen), MAP2 (Anti-Map2 Mouse, Invitrogen), and PSD95 (Anti-PSD95 rabbit, Invitrogen) for neurons. To identify the stages, we plated the cells onto German cover slips (18 mm) coated with appropriate substrates (Cho et al., 2011). Approximately 5×10^4^ cells were plated per coverslip at each stage for immunofluorescent analysis. 4% paraformaldehyde (5–10 min) was used for fixation, followed by 1X phosphate-buffered saline (PBS) washes. Fixed cultures were kept for 1 h at room temperature in freshly prepared blocking solution in 1X PBS with 1% (w/v) bovine serum albumin (BSA; 0.3% (v/v) Triton X-100 and 0.05%) sodium azide and then treated with stage-specific primary antibodies prepared in the blocking buffer, overnight at 4°C. Next, secondary antibodies were applied at 1:500 dilution for 60 min at room temperature in the dark. A 1 mg/mL 4′,6-diamidino-2-phenylindole (DAPI, Life Tech, 62,248) stock solution was diluted in PBS (1:1,000) before adding to the wells to stain the nuclei. After 10 min, the solution was washed off twice with PBS before mounting the coverslips on glass slides with the anti-fading reagent (Molecular probes, P36930). The cells were visualized at 20x magnification using Zeiss inverted fluorescence microscope (Zeiss 710 microscope).

### 2.4 Nuclear magnetic resonance spectroscopy (NMR)

Proton NMR spectra (1H-NMR) were generated for each cell type using the 800 MHz NMR spectrometer with cryoprobe (Avance; Bruker, Inc.) to assess specific metabolome profiles in differentiating cells. NMR was acquired on 1,000,000 cells for each population. Cells were washed with PBS and spinned down at 1,000 g for 1–2 min at 4°C to remove the remnant culture media (Ma et al., 2011). The pellet was resuspended in 450 μL of PBS, pH 7.0, in D_2_O and 50 μL of 50 mM internal standard 2,2-dimethyl-2-silapentane-5-sulfonate (DSS) in D_2_O. To examine the cell metabolome, samples were sonicated and kept at 4°C if data acquisition was within 3–4 h or at −80°C if data acquisition was later. Sonication breaks up the cells and allows the metabolites to exit into the PBS, increasing their mobility in the NMR field (Singh et al., 2023; Mielko et al., 2021; Leibel et al., 2022). All experiments were done in triplicate within the same culture (i.e., on the same day) to ensure reproducibility, and with 3–8 biological replicates for specificity. 1H-NMR 1D spectra were acquired for 30 min at 25°C. Before Fourier transform, FIDs were line-broadened to 1.0 Hz with an exponential weighting function, phase and baseline corrected for distortions and referenced to DSS chemical shift. To identify the J-coupled peaks that belong to the same molecule, 2D NMR was acquired for 24 h. 2D, 1H-1H NMR spectra (Total Correlation Spectroscopy (TOCSY)) was collected at 25°C. The pulse sequence dipsi2esgpph (homonuclear Hartman-Hahn transfer) was selected. Excitation sculpting with gradients was applied for water suppression. The spectral width was 13.95 ppm and the number of scans was 24. Spectra were pre-processed using TopSpin (Bruker, Inc.) and uploaded to Chenomx, Inc. software (Chenomx Inc., Edmonton, Canada) for peak identification. Chenomx has an 800 MHz library consisting of 380 metabolites, which is manually queried for each sample. We cross-referenced the spectral signature of each metabolite to the Biological Magnetic Resonance Bank (BMRB) public database.

(BMRB) public database. Upon complete query, a list of small molecules in the sample and their estimated concentration (based on the integrated area under the peaks) was derived (*n* = 90) (Supplementary Table S1). The identities of molecules were cross validated using 2D NMR data (Arnold et al., 2015).

### 2.5 Computational methods and software package

As copious biological network data continues to be gathered in various omics domains, standardized methods which process complex network information into interpretable and visualizable representations can help to turn diverse datasets into novel insights. Toward this goal, we have used the combination of PCA analysis and GLASSO to analyze the relative contribution of all features in the NMR datasets and infer their relative networks. PCA is a statistical method that reduces the dimensionality of a dataset by calculating linear combinations of features which maximally capture the variance of the dataset (Gewers et al., 2022). The few dimensions that explain most of the variance (the principal components) are frequently isolated and used to cluster the data in a visualizable lower-dimensional space. Analyzing the relative contribution of each feature towards each PCA dimension can suggest a relationship between the features and samples which score highly along that dimension. When run over the full dataset, with samples from each of the five neural cell populations, PCA clearly separates the cell types in an unsupervised manner, but due to the five-dimensional classification of cell types, it is unclear how to associate even the most outlying features with specific cell types. By instead running PCA multiple times over each sequential cell-type pair, we produce a binary classification setup that allows the association of individual features (metabolites) with one of the two cell types. However, the PCA scores of most features cluster tightly around 0, and only the most outlying of the metabolites can be confidently associated.

In turn, GLASSO applies L1-regularization to the covariance matrix to produce a sparse covariance matrix. Producing a sparse matrix is especially valuable in higher-dimensional settings, like those of metabolomics, where a naive covariance matrix would yield too many connections to be easily interpretable. We used GLASSO on the full dataset (agnostic of cell type) to generate a sparse covariance network that reduces the noise of metabolome data, yielding a visually interpretable network of feature correlations revealing metabolome structure (Friedman et al., 2008; Mazumder and Hastie, 2012). This method captures both positive and negative correlations, while leaving some features with no edges removed from the graph.

Overlaying the GLASSO network with PCA scores allows the PCA scores of individual features to be placed within the context of the feature covariance relationships.

We use the PCA and GLASSO implementations from *scikit-learn*,^1^ found in the *sklearn.covariance* packages, respectively. All plotting is done using *matplotlib*,^2^ and data I/O makes use of *pandas.*^3^ All code is run with python version 3.7.12. The class ‘pca_bundle’ runs PCA and allows the easy visualization of PCA-related graphs, showing loadings, scores, and explained variance. All plotting functionality leverages the matplotlib package.

### 2.6 Implementation

We ran PCA over the mean- and stddev-normalized dataset to produce 10 components, of which we use the first three. When applied to the entire dataset, GLASSO is run with an alpha selected by cross-validation via the *sklearn.covariance.GraphLassoCV* implementation (alpha = 0.56, 250 iterations), using the default values for the cross-validation procedure. When applying GLASSO to each cell-transition pair, we set the alpha to 0.9, as the truncated datasets for each pair are small and thus unstable under cross-validation.

### 2.7 Code

The MetaboLINK code is publicly available.^4^ Additionally, we make publicly available an interactive python notebook through Google Colab, which walks through the application of PCA and GLASSO as well as their various visualizations. We hope this will facilitate the easy use of MetaboLINK for novel applications.

### 2.8 Metabolomic pathway analysis

The metabolic maps and pathways derived from MetaboLINK analysis were manually generated by combining existing KEGG metabolic pathways (KEGG, n.d.) and cross-matching HMDB database (Human Metabolome Database, n.d.). MetaboAnalyst software (Xia and Wishart, 2016)^5^ has also been used for correlation analysis, Partial Least Squares—Discriminant Analysis (PLS-DA) and Pattern Hunter studies. Correlation analysis was used to visualize the overall correlations between different features. With PLS-DA, we calculated the Variable Importance in Projection (VIP) that is a weighted sum of squares of the PLS loadings considering the amount of explained Y-variation in each dimension. Furthermore, Pattern Hunter functionality in MetaboAnalyst was applied to enable the identification of metabolites that follow a predefined pattern of concentration changes. We applied this module to identify patterns for all metabolites across different developmental stages. The pattern to be tested needs to be specified as a series of numbers separated by “-” where each number describes the expected relative concentration change at the corresponding sampling point. Thus, a pattern designated as “1–2–3-4-5” would search for metabolites with linearly increasing values across the five corresponding sampling points (hESC-EB-Rosettes-hNPC-Neurons), whereas a pattern defined as “2–1–1-1-1” and “1–1–1-1-2” would describe a specific increase and decrease in just hESC and neurons, respectively.

## 3 Results

### 3.1 PCA-GLASSO identifies diverse metabolites in distinct neural lineages

**Figure 1:**
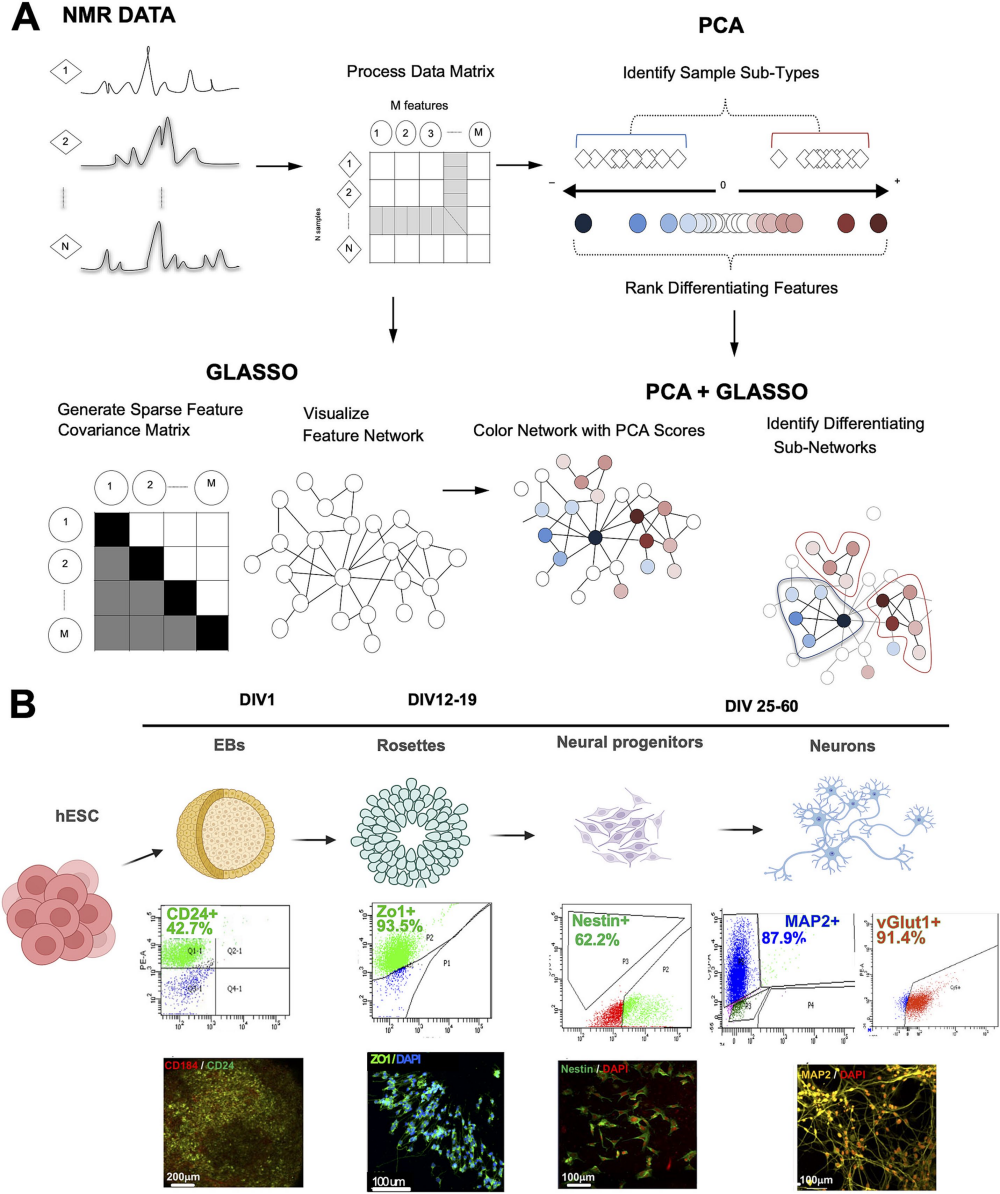
The principle behind the design of the PCA-GLASSO analysis method applied to complex metabolic human neuronal differentiation data. **(A)** Analysis of the metabolome data. Proton NMR spectra generated for any given sample are organized into a corresponding matrix of samples and features. The matrix is employed in either PCA or GLASSO analysis, where each technique provides a level of insight into the metabolome at hand (PCA: identifies sample subtypes with ranked differentiation features; GLASSO: identifies network of samples and features). Combined PCA-GLASSO analysis identifies differentiating subnetworks in PCA-clustered samples. **(B)** Schematic representation of human neuronal differentiation from hESCs. The cascade of cell types includes embryonic bodies (EBs), human neural progenitors (hNPCs), and neurons. Cell-type specific markers were used to identify each one of the cell types. The purity of stage-specific lineages was evaluated by flow cytometry. Immunocytochemistry was performed to characterize each stage of differentiation. PCA: principal component analysis, GLASSO: graphical lasso, hESC: human embryonic stem cells; NMR: nuclear magnetic resonance spectroscopy. Figure created using Biorender.

To integrate multivariate data analysis, such as PCA, with the statistical network visualization conferred by GLASSO, we designed a new algorithm combining the two analytical approaches, PCA-GLASSO (Figure 1A). The algorithm processes high-dimensional datasets, including longitudinal metabolome data. To test the algorithm, we used hESCs that underwent induction and differentiation into four distinct neural lineages: EBs, rosettes, hNPCs, and neurons (Figure 1B). To ensure cell type specificity prior to NMR metabolomics profiling, we performed flow cytometry to examine cell type purity at each stage of differentiation (Figure 1B). For EBs and rosettes, we used surface markers such as CD24 and ZO-1, respectively. For neuroprogenitors, we used nestin, an intracellular marker, and for neurons, we used both MAP2 and vGLUT1 to ensure we had glutamatergic neurons in the culture (Figure 1B). Based on the flow cytometry data, we sorted EBs and plated sorted cells to induce their differentiation into rosettes. No other cell types were sorted, but at different stages we dissociated and plated onto new coverslips/wells to move forward along the differentiation cascade to neurons. Based on flow cytometry data, EB and hNPCs were least pure, thus containing a mixture of cells, and rosettes and neurons were mostly pure. In addition to flow cytometry, at each stage we performed immunocytochemistry to determine the expression of cell type-specific markers (Figure 1B).

**Figure 2:**
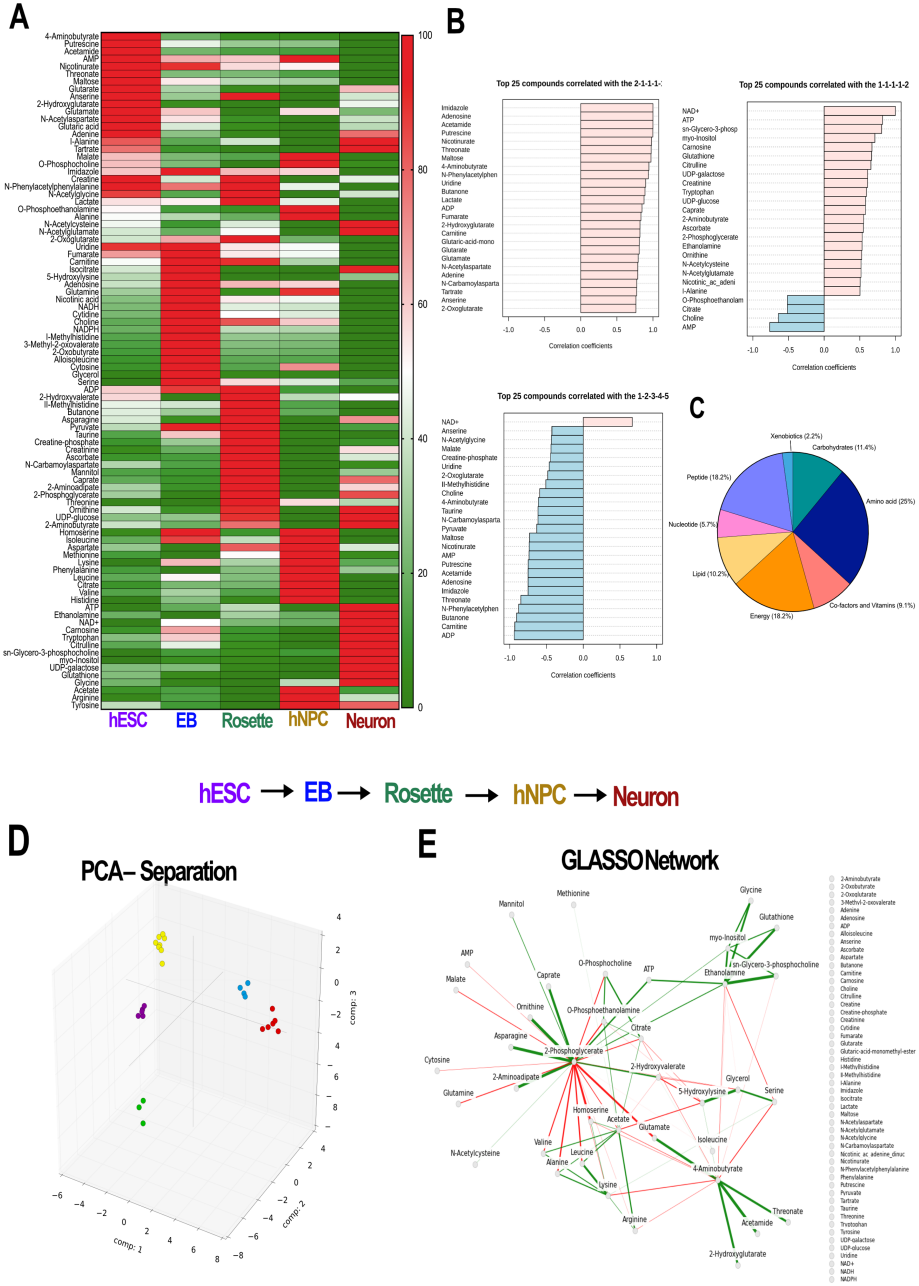
Different cell types along the neuronal differentiation cascade have distinct metabolic signatures. **(A)** Heat map of the metabolite normalized abundance identified by NMR analysis. Each cell type is enriched in a specific set of metabolites. **(B)** Selected examples of pattern hunter profiles represent metabolites and their match with the tested pattern. Each dataset shows both positively correlated (pink) and negatively correlated (blue) metabolites with pattern group 2–1–1-1-1 (hESC-EB-Rosette-hNPC-Neuron) or pattern group 1–1–1-1-2 (hESC-EB-Rosette-hNPC-Neuron) show the metabolites following higher distribution in hESCs and neurons, respectively. Bottom panel: metabolites with linearly increasing values across the five corresponding sampling clusters negatively correlate for a pattern 1–2–3-4-5, except for the NAD+ (pink). **(C)** Pie chart of all metabolites shows distribution of 8 classes of metabolites in all samples, with the largest contribution from amino acids. **(D)** 3D plot of the first three components of the PCA shows clear separation of the cell types. Each circle is a sample. **(E)** GLASSO-generated sparse covariance network shows positive (solid) and negative (dashed) correlations between metabolites. PCA: principal component analysis, GLASSO: graphical lasso, hESCs: human embryonic stem cells; EB: embryonic bodies, hNPCs: human neuroprogenitors; NMR: nuclear magnetic resonance spectroscopy.

At each stage of differentiation, we collected 1 million cells and performed NMR. The biologically different hESC-derived neural progenies had very different NMR spectra (Supplementary Figure S1), pointing at the unique metabolome at each lineage. Chenomx analysis (Chenomx, Inc.), a widely used software to identify metabolites in the NMR spectra, detected a total of 90 metabolites among the cell types. Interestingly, we could separate each cell stage based on the abundance of metabolites (Figure 2A), even though our EB and hNPC populations were not completely pure (Figure 1B). Globally, in hESCs we identified high abundance of glutamate-derived metabolites while glutathione, ATP, and NAD+ were more abundant in the neuronal cluster (Figure 2B). We observed direct linear reduction across different neural stages for ADP, AMP, adenosine, and carnitine (Figure 2B, Supplementary Figure S2A), with high concordance with nicotinamide-related metabolites (Supplementary Figure S2B). Among all the metabolites identified, amino acids represented 25% of the metabolites, while bioenergetics, peptides, and carbohydrates accounted for 18.2, 18.2, and 11.4%, respectively (Figure 2C).

**Figure 3:**
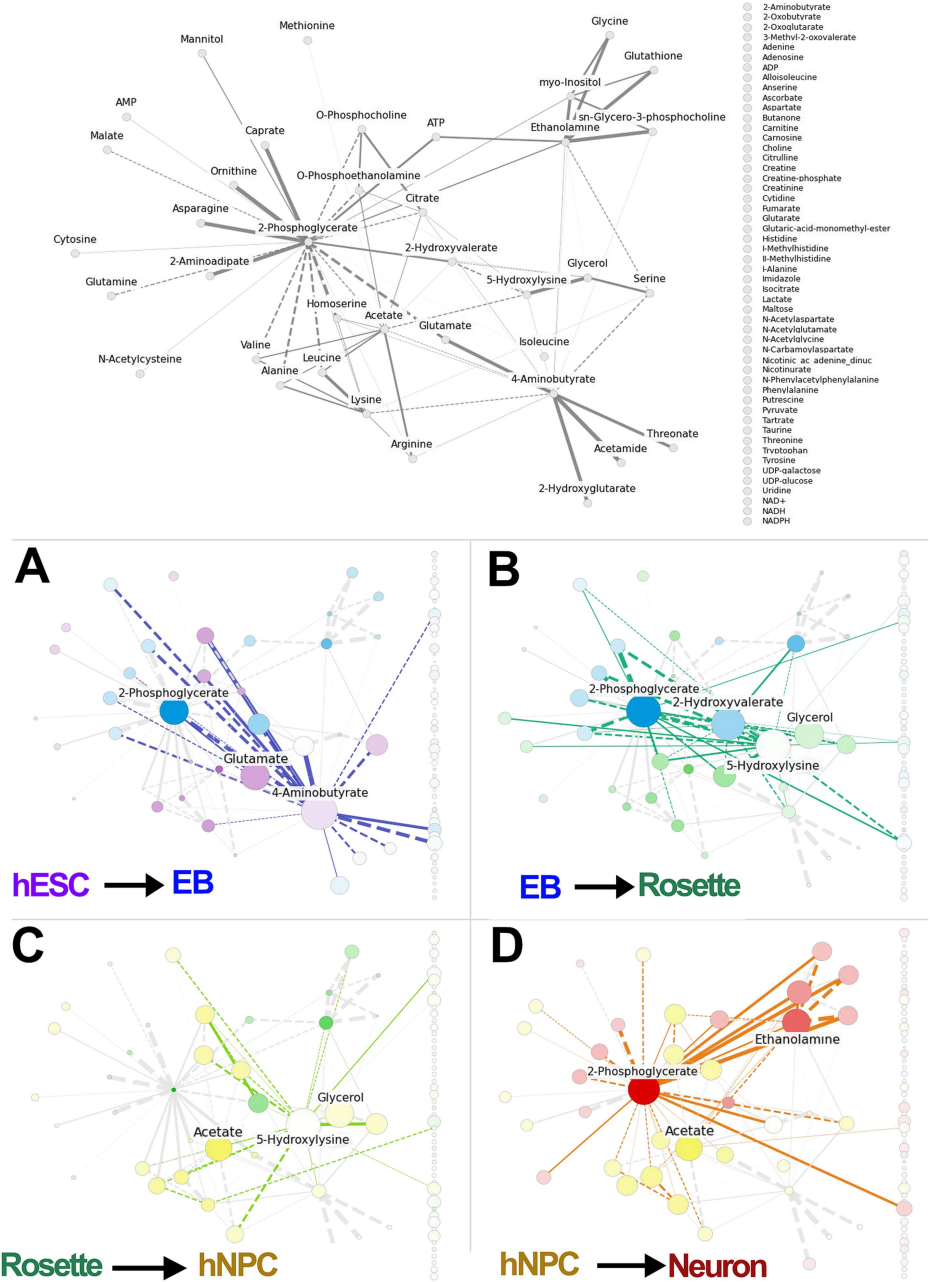
Integration of PCA-GLASSO analyses identifies metabolic pathways specific for each differentiation stage. The full GLASSO network for all samples (same as Figure 2E). GLASSO alone identifies the structure of the metabolomic network but offers no insight into any relationship between individual metabolites and different cell types. **(A–D)** Integrated PCA-GLASSO (i.e., MetaboLINK) applied to different cell transitions identifies metabolic hubs within the global metabolic network map that selectively dominate a given transition. The node sizes reflect the magnitude of change in concentration of each metabolite over the transition. The color intensity of each node reflects the magnitude of the PCA score for that metabolite, and the color itself shows which cell type the metabolite is associated with. Overall, this combined information of the total network structure with the individual transition GLASSO network, PCA scores, and change in concentrations, makes it clear which metabolites and subnetworks of metabolites are implicated in each cell transition. PCA: principal component analysis, hESCs: human embryonic stem cells, EB: embryonic bodies, hNPC: human neuroprogenitors.

To identify and rank differentiating features, we used PCA on detected metabolites from the developing neural cell lineages. This was followed by GLASSO analysis to generate a sparse feature covariance matrix and visualize feature networks. The PCA confirmed the distinct cell populations by clustering each cell lineage separately (Figure 2D). GLASSO, in turn, revealed a distinct network of sample features identifying clusters and sub-clusters among the samples analyzed (Figure 2E). While GLASSO alone revealed the structure of the metabolomics network, it did not provide insights into the relationships between individual metabolites and cell types. Therefore, we combined PCA with GLASSO to assess the relative contributions of all features in our longitudinal metabolomics dataset and to infer complex, interrelated networks among features (metabolites) (Figures 1A, 3). Subsequently, the metabolite nodes in the GLASSO network were colored based on PCA loading scores associated with cell-type transitions (see Figures 2D, 3 top panel). Each node represents a chemical compound, while the edges (lines) signify the set of reactions linking these metabolites. The network is depicted using solid lines indicating positive correlations and dashed lines representing negative correlations; the thickness of these lines reflects the strength of the interactions. Transition-specific PCA scores and the GLASSO network for each of the four cell-type transitions are overlaid on the full GLASSO network, shown in grey background (Figures 3A–D). The transition-specific networks are color coded per cell type, and the most significant metabolites are labeled for clarity. Node sizes indicate the magnitude of concentration changes for each metabolite during the transition, while the color intensity reflects the magnitude of the PCA scores. The color itself denotes the specific cell type with which the metabolite is associated. This visualization yields a graphic with a straightforward interpretation: similarly colored metabolites that cluster within the network are highlighted as functionally related sub-networks. This comprehensive visualization seamlessly integrates the overall network structure with individual transition scores and variations in metabolite concentrations. By doing so, it effectively highlights which metabolites and their associated subnetworks are implicated in each cell transition. The overarching network structure, presented in a neutral grey, establishes a reference framework that allows for easy comparison against the transition-specific networks, which are distinctly color-coded to represent their unique characteristics. This color distinction not only facilitates identification of the different transitions but also illustrates the specific cell types linked to each metabolite as well as functionally related sub-networks, enhancing interpretability and clarity.

By integrating insights from PCA with the metabolite network structure derived from GLASSO into a unified algorithm, MetaboLINK, this approach facilitates a network-level analysis of transitions between paired cell types. Notably, we identify key metabolites such as glutamate, 4-aminobutyrate, and 2-phosphoglycerate as components of the predicted subnetwork involved in the transition from hESCs to EBs (Figure 3A). These findings highlight the central role these metabolites may play in facilitating the transition process from hESCs to EBs, and potentially uncovering underlying metabolic shifts and differential cell-specific metabolic demand.

In the subsequent section, we focus on validating the biological significance of the networks highlighted by the MetaboLINK strategy, further exploring the functional implications of these key metabolites and their interactions within the metabolic landscape. This validation will enhance our understanding of how these networks contribute to cellular transitions and their broader biological contexts.

### 3.2 Amino acids, lipids, and bioenergetic pathways distinguish different stages of neural cell differentiation

To navigate and interpret the intricate metabolic landscape of the neural cell types identified by the MetaboLINK for a deeper understanding of neural development and function, we highlight several biologically significant pathways that have emerged from the data.

**Figure 4:**
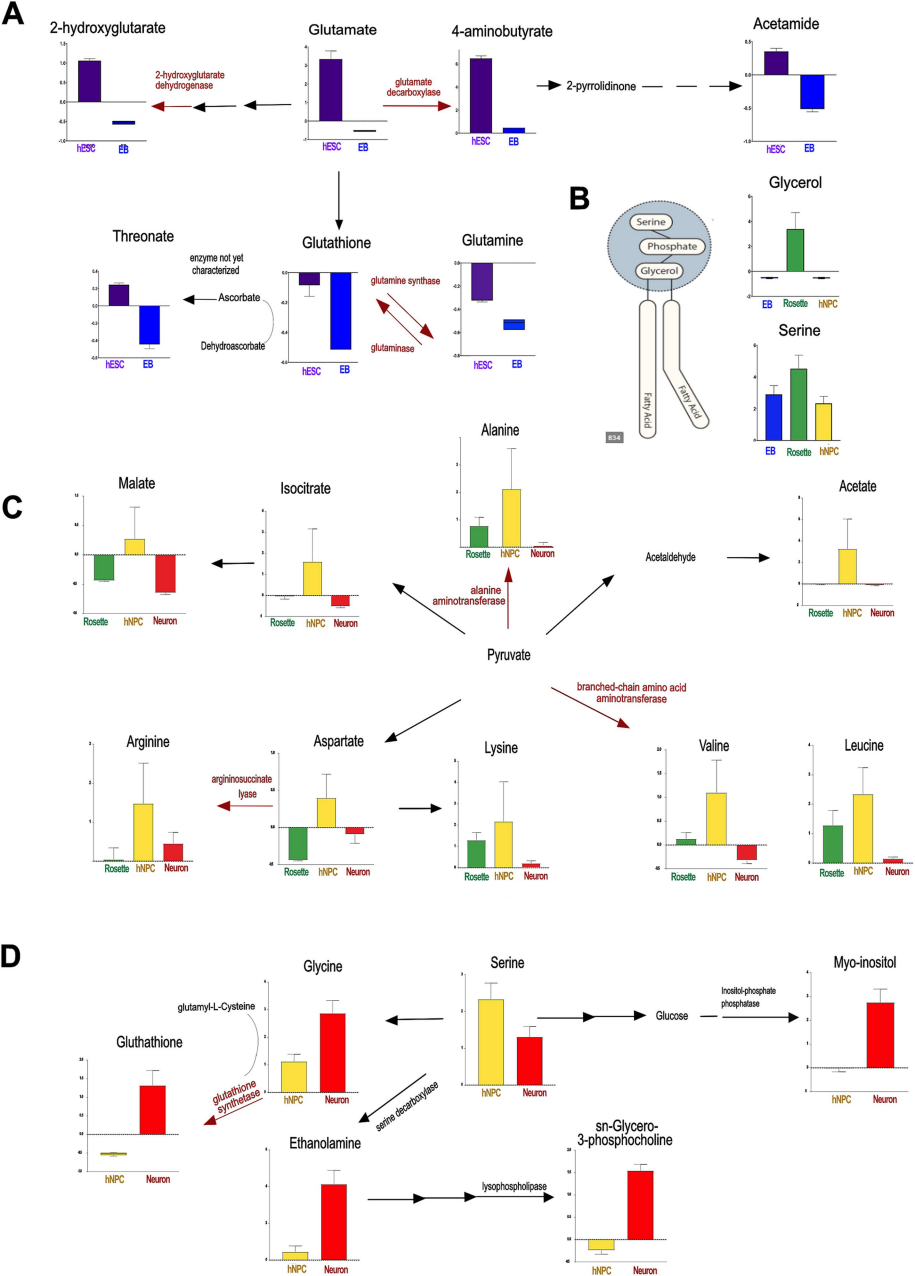
The metabolic pathways identified by MetaboLINK show prevalent networks at cellular transition states to neurons. The enzymes associated with the conversion of key metabolites are showed in red along with the associated human disease. **(A)** The principal metabolic shift, occurring in the transition from hESCs to EBs, is in glutamate-derived metabolism. The dotted arrow shows potential novel enzymatic conversion. **(B)** In Rosettes, the highest concentration of glycerol and serine, the main components of phosphatidylserines, as well as arginine-related metabolites are significant drivers. **(C)** In hNPCs, principal modifications are dibasic and branched chain amino acids. **(D)** In neurons, key components of glycerophospholipids metabolism and scavengers showed significantly higher values. The gene encoding enzymes associated to diseases are reported below: Glutamate decarboxylase, GAD1, Developmental and epileptic encephalopathy 89 (AR, #OMIM 619124); 2-hydroxyglutarate dehydrogenase, L2HGDH, L-2-hydroxyglutaric aciduria (AR, #OMIM 236792); Alanine aminotransferase 2, GPT2, Neurodevelopmental disorder with microcephaly and spastic paraplegia (AR, #OMIM 616281); Argininosuccinate lyase, ASL, Argininosuccinic aciduria (AR, #OMIM 207900); Glutathione synthetase, GSS, Glutathione synthetase deficiency (AR, #OMIM 266130). hESCs: human embryonic stem cells, EB: embryonic bodies, hNPCs: human neuroprogenitors. *p < 0.05, **p < 0.01, ***p < 0.0001.

First, MetaboLINK analysis of the NMR-identified metabolome of neural cells found higher abundance of glutamate and GABA in hESCs compared to other committed lineages (Figure 4A and Supplementary Figure S5). Glutamate and its related metabolite GABA are essential for brain function and neural development through several key mechanisms. Glutamate is a precursor in glutathione synthesis that contributes to maintaining cellular redox balance by influencing glutathione levels. Glutathione maintains neural redox balance, influencing reactive oxygen species (ROS) levels and progenitor cell proliferation (Renault et al., 2009). Additionally, glutamate serves as an energy source; it is metabolized to *α*-ketoglutarate by glutamate dehydrogenase, participating in the citric acid cycle and thereby aiding ATP synthesis, which is crucial for neuronal cell fate and mitochondrial function (Homem et al., 2014; Tohyama et al., 2016; Watkins, 2000). Mutations in genes associated with glutamate metabolism play a role in developmental disorders such as developmental and epileptic encephalopathy (MIM: 618328, 618,412), underscoring the critical role of maintaining proper glutamate levels for normal brain function (Neuray et al., 2020). The increased glutamate signaling in hESCs may result from enhanced uptake or reduced degradation. Undifferentiated hESCs exhibit highly efficient glutamine-to-glutamate conversion regulated by highly expressed mitochondrial glutaminase (*GLS2*) (Marsboom et al., 2016; Hensley et al., 2013). The efficient conversion of glutamine to glutamate by *GLS2* in hESCs declines upon differentiation (Marsboom et al., 2016), and this may contribute to the highest glutamate level observed in hESCs.

Additionally, rosettes exhibited elevated levels of glycerol and serine, which are key components of phospholipids (Figure 4B). Glycerol serves as the backbone for phospholipid molecules, while serine contributes to the synthesis of phosphatidylserine and other essential phospholipids. The increased presence of these metabolites in rosettes highlights their critical role in membrane biosynthesis and cellular structure within these neural cell aggregates, as previously suggested (Arai et al., 2015; Sánchez-Ramírez et al., 2024).

In turn, hNPCs showed higher concentrations of branched-chain amino acids (BCAA) valine and leucine, supporting adaptive changes in BCAA metabolism for an energy shift (Figure 4C, Supplementary Figure S4) (Bifari et al., 2020; Abdi et al., 2022). BCAA metabolism is critical for learning and memory, hippocampal neurogenesis, and neuronal differentiation, underscoring its critical role in cognitive function and neural development (Abdi et al., 2022). Furthermore, hNPCs had increased content of arginine and related metabolites compared to other neural cell types (Geiger et al., 2016). Arginine induces a metabolic switch from glycolysis to oxidative phosphorylation (OXPHOS), which is highly relevant for neurogenesis (Iwata et al., 2023; Kondoh et al., 2010).

Finally, neurons exhibited higher levels of specific lipids, such as sn-glycerol-3-phosphocholine, crucial for membrane build-up and signaling compared to other cell types (Figure 4D and Supplementary Figure S6). Sn-glycerol-3-phosphocholine, an intermediate in glycerophosphocholines-derived phospholipids like phosphatidylcholine, is significantly abundant in mature neurons. Beyond their structural role in membranes, these lipids act as bioactive molecules, impacting prostaglandin pathways, peroxisome proliferator-activated receptors responses, and more generally, lipid metabolism (Furse and de Kroon, 2015). The pro-neurogenic effect of phosphatidylcholine (Magaquian et al., 2021; Kuge et al., 2014) aligns with our findings, emphasizing the necessity of precise control over mature neurons membrane composition. Furthermore, ethanolamine, a phospholipid component, was higher in neurons compared to other cell types, potentially influencing neurogenesis especially under inflammatory stress (Montaner et al., 2018; Magaquian et al., 2021) (Figure 4D). Finally, neurons exhibited elevated levels of glutathione and myo-inositol (Figure 4D and Supplementary Figure S2C), both known as ROS scavengers, supporting their role to counterbalance ROS generated during OXPHOS-ATP production (O’Brien et al., 2015).

Among the various metabolic hallmarks, a notable shift in bioenergetics was observed as cells transitioned from hESCs to mature neurons (Supplementary Figure S3). The metabolic fate of neuroprogenitors is intricately linked to a metabolic rheostat governing mitochondrial dynamics, an evolutionary conserved phenomenon from flies to humans (Bonnefont and Vanderhaeghen, 2021; Iwata and Vanderhaeghen, 2021). ATP generation pathways, such as glycolysis and mitochondrial OXPHOS, play a critical role in fulfilling the distinct energetic demands of hESCs versus the needs of differentiating progenies (Folmes et al., 2012; Becker et al., 2010; Birket et al., 2011; Zhang et al., 2014). Glycolysis, although less ATP-efficient than OXPHOS, is faster and suitable for scenarios demanding quick energy bursts, making it advantageous for immature or rapidly proliferating cells like hNPCs (Seo et al., 2018; Tohyama et al., 2016; Armstrong et al., 2010). Additionally, glycolysis supports overall cellular growth and proliferation. Conversely, mitochondria respiration through OXPHOS stands out as the most efficient pathway for ATP generation, which is the preferred mechanism for cells with high energy demands such as neurons (O’Brien et al., 2015; Lange et al., 2016; Ito and Suda, 2014; Roy et al., 2018; Iwata and Vanderhaeghen, 2021). The balance between these pathways is crucial in mitigating mitochondrial ROS production (Stacpoole et al., 2011) and meeting cell-specific energy demands across different developmental stages. We observed higher concentrations of AMP, ADP, lactate, pyruvate, and alanine in hESCs. Conversely, neurons displayed the highest levels of ATP and NAD+, indicating a transition towards OXPHOS activity (Figure 2A and Supplementary Figure S2A, S3). Increase in NAD+ (oxidized form of NADH) to NADH ratio activates a NAD + -dependent deacetylase to inhibit hNPC vs NPC self-renewal and promote their differentiation (Hisahara et al., 2008). Our data support a model in which glycolysis sustains stemness, while the transition to OXPHOS is closely linked to neuronal differentiation (Agathocleous et al., 2012; Zheng et al., 2016; Bifari et al., 2020).

## 4 Discussion

Neurogenesis, a pivotal event in neural development, marks the transition of neuroprogenitor cells from self-renewing entities to post-mitotic neurons. While intrinsic and extrinsic cues play a central role in guiding cellular transitions (Iwata and Vanderhaeghen, 2021; O’Neill et al., 2016), the involvement of metabolic pathways remains less explored. In this context, metabolomics emerges as a high-throughput and sensitive platform offering a unique lens to understand how metabolism governs undifferentiated progenitors and their differentiated neural progenies. Navigating the complex ‘omics datasets demands sophisticated bioinformatic tools and metabolomics is no exception. Indeed, metabolomic analysis is often hindered by the lack of efficient tools for identifying key metabolic hallmarks and understanding complex biological networks.

To address these issues, we introduce a novel approach, MetaboLINK, to facilitate metabolite discovery that not only discriminates cell types—identified by PCA clustering—but also contributes to functional networks when visualized through GLASSO. Overlaying the GLASSO network with PCA scores allows the PCA scores of individual features to be placed within the context of the feature covariance relationships. This integration can reveal subnetworks within the data that might remain hidden while using PCA or GLASSO analysis separately. It also provides insightful interpretations for the majority of PCA scores, which might otherwise be too subtle to interpret clearly on their own. Thus, insights from PCA that highlight individual metabolites are integrated with the metabolite network structure derived from GLASSO, facilitating a network-level analysis of the transitions between paired cell types. This combined approach allows for a deeper understanding of the metabolic changes and interactions occurring during these transitions. It allows us to understand how several metabolites belonging to the same pathway interact within a given cellular lineage, gain a more comprehensive view of metabolites roles across different cell types, and enhance our understanding of the overall metabolic landscape. MetaboLINK also effectively contextualizes low-scoring features, providing insights into underlying biological networks and supporting its applicability and feasibility in other big data analysis scenarios.

MetaboLINK identified specific metabolites and related hidden interrelationships across diverse neural stages. Validation of these metabolites and their related pathways through existing pathway databases ascertained the robustness of the uncovered networks and the biological significance of MetaboLINK-discovered enrichments. Namely, MetaboLINK analysis revealed distinct hallmarks in glutamate, lipid, and energy metabolism in specific neural developmental stages, which aligned to specific cellular functions. For instance, changes in glutamate metabolism in hESCs reflect adjustments in ROS balance and oxidative stress to maintain redox equilibrium during maturation. Fluctuations in lipid metabolism in hNPCs and neurons could be linked to alterations in cellular membrane composition and signaling pathways. Additionally, variations in energy metabolism in neurons highlight shifts in bioenergetic demands as neuronal cells mature.

The identification of novel metabolic signatures along the neuronal differentiation cascade through MetaboLINK could advance our understanding of neurodevelopmental disorders. For example, when we queried MetaboLINK pathways-related enzymes we identified already well-known human neurodevelopmental and neurological disorders. Disruptions in the enzymes associated with glutamate, lipid, and bioenergetics, as identified by MetaboLINK, underscore the essential role of these pathways in developing brain. Imbalances in glutamate-related enzymes affect neurotransmission, and this may contribute to autism spectrum disorders and intellectual disabilities (Montanari et al., 2022). Abnormalities in enzymes of lipid metabolism can affect neuronal membrane integrity and signaling, contributing to disorders such as multiple sclerosis (López-Muguruza and Matute, 2023). Similarly, disruptions in enzymes involved in ATP generation—impairing cellular energy homeostasis—can lead to mitochondrial diseases and related neurodevelopmental diseases (Clemente-Suárez et al., 2023). These signatures might reveal new pathways and mechanisms involved in disease onset and progression, thereby offering fresh perspectives on the underlying causes of such disorders and potentially leading to more effective therapeutic interventions.

The application of MetaboLINK not only refines our comprehension of metabolomic landscapes in different stages of neurogenesis but also paves the way for strategies (metabolic or pharmacological interventions) aimed at modulating stem cell fate and consequently brain development and functionality. A deeper understanding of specific metabolic signatures in distinct cell populations is essential for targeted *in vitro/in vivo* approaches to direct undifferentiated stem cells towards desired cellular phenotypes (Martano et al., 2019). Supplementation with antioxidants, BCAAs, and phosphatidylcholine might hold promise for enhancing neuronal maturation.

MetaboLINK analysis of omics datasets adds structure to complex biological data, aiding data interpretation and hypothesis generation with clarity. Its applicability stretches beyond the boundaries of metabolomics, as it can be modified for any domain dealing with complex network information, be it biological or otherwise. To catalyze the broader adoption of the MetaboLINK analytical algorithm, we contribute the code employed in this study to the public domain, fostering collaboration and accelerating advancements in diverse scientific disciplines.

## Data Availability

The datasets presented in this study can be found in online repositories. The names of the repository/repositories and accession number(s) can be found in the article/supplementary material.

## Footnotes

^1^ https://scikit-learn.org/stable/version.0.22.2.post1

^2^ https://matplotlib.org/version.3.2.2

^3^ https://pandas.pydata.org/version.1.1.5

^4^ https://github.com/jlichtarge/pcaGLASSO

^5^ https://www.metaboanalyst.ca/MetaboAnalyst/

## Supplementary material

SUPPLEMENTARY FIGURE S1 NMR spectra of different cell types along the differentiation cascade from hESCs to neurons. Each cell type has a unique spectral signature that denotes different metabolome make up of these cells. NMR: nuclear magnetic resonance spectroscopy.

SUPPLEMENTARY FIGURE S2 **(A)** Important features identified by PLS-DA. The colored boxes on the right indicate the relative concentration of the corresponding metabolite in each group. **(B)** The overall correlation heatmap shows high correlation among purine-related metabolites (especially bioenergetic molecules) and nicotinamide-pathway related metabolites. **(C)** Specific distribution of glutathione across different cell-population shows higher abundance in neurons vs the rest of the samples.

SUPPLEMENTARY FIGURE S3 ATP levels are significantly higher in neurons supporting the high energy rate (and OXPHOS-dependence) in these cells.

SUPPLEMENTARY FIGURE S4 TCA-related metabolites are more abundant in hESCs and neurons vs the other clusters, potentially related to their high energetic demands and substrates. Also, hESCs show the highest level of lactate and pyruvate, proving the high glycolytic state for these cells.

SUPPLEMENTARY FIGURE S5 Glutamate-related metabolites are shown for all the different groups (hESCs, EBs, rosettes, hNPCs, and neurons). In particular, high levels of glutamate and GABA were identified in hESCs vs the other clusters, while glutathione and creatinine levels were more abundant in neurons.

SUPPLEMENTARY FIGURE S6 High levels of glycerophophocholine were identified in neurons, while intermediate substrates like phosphoethanolamine and phosphocholine are more abundant in early precursors.

SUPPLEMENTARY TABLE S1 Normalized abundance of 90 NMR-derived metabolites at each cellular stage is provided.
