# Supplementary figures and images for: MetaboLINK is a novel algorithm for unveiling cell-specific metabolic pathways in longitudinal datasets
Source: Front Neurosci. 2025 Jan 13;18:1520982. doi: 10.3389/fnins.2024.1520982 (PMC11769959; doi:10.3389/fnins.2024.1520982)

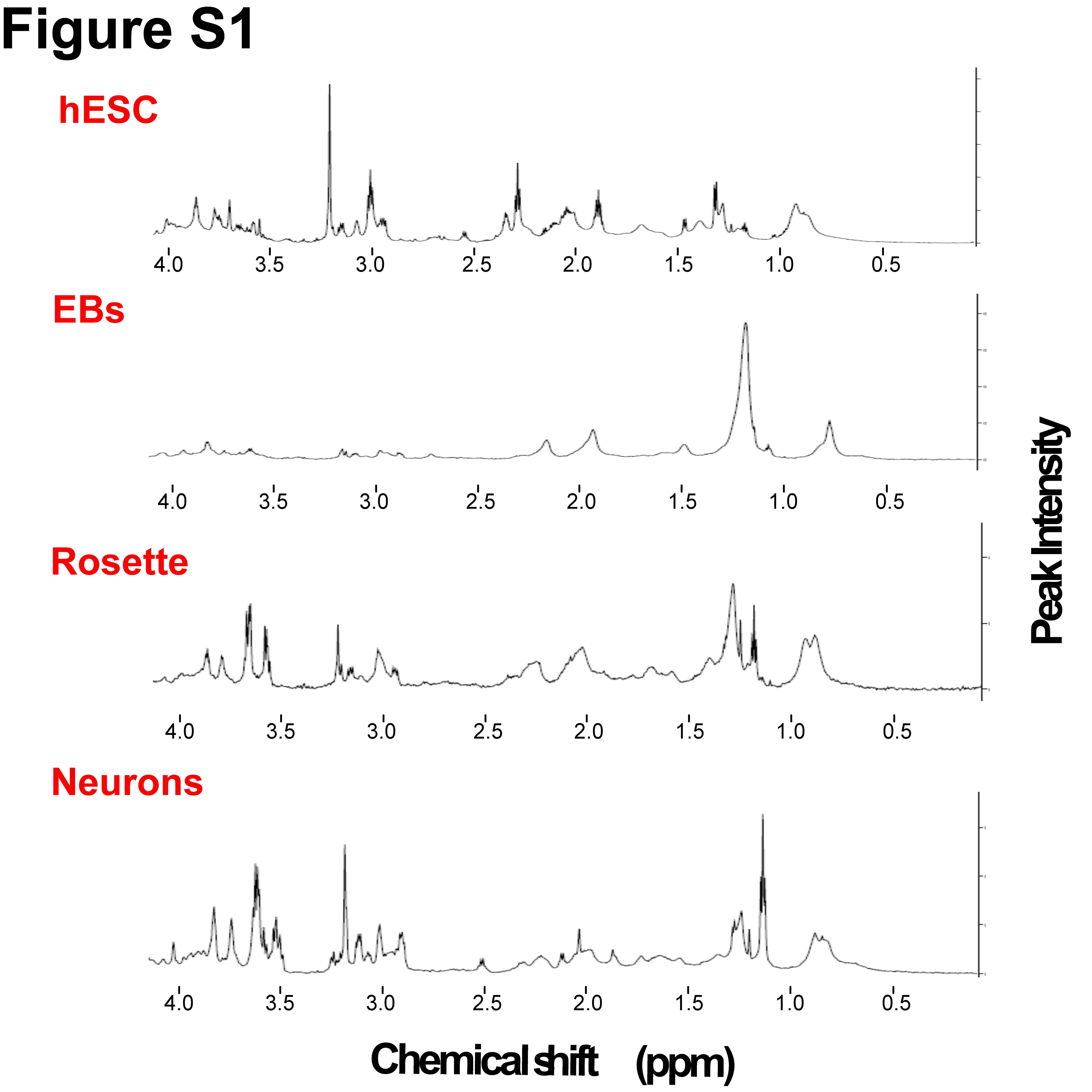

Supplement: SUPPLEMENTARY FIGURE S1 — NMR spectra of different cell types along the differentiation cascade from hESCs to neurons. Each cell type has a unique spectral signature that denotes different metabolome make up of these cells. NMR: nuclear magnetic resonance spectroscopy. [file Image_1.jpeg]

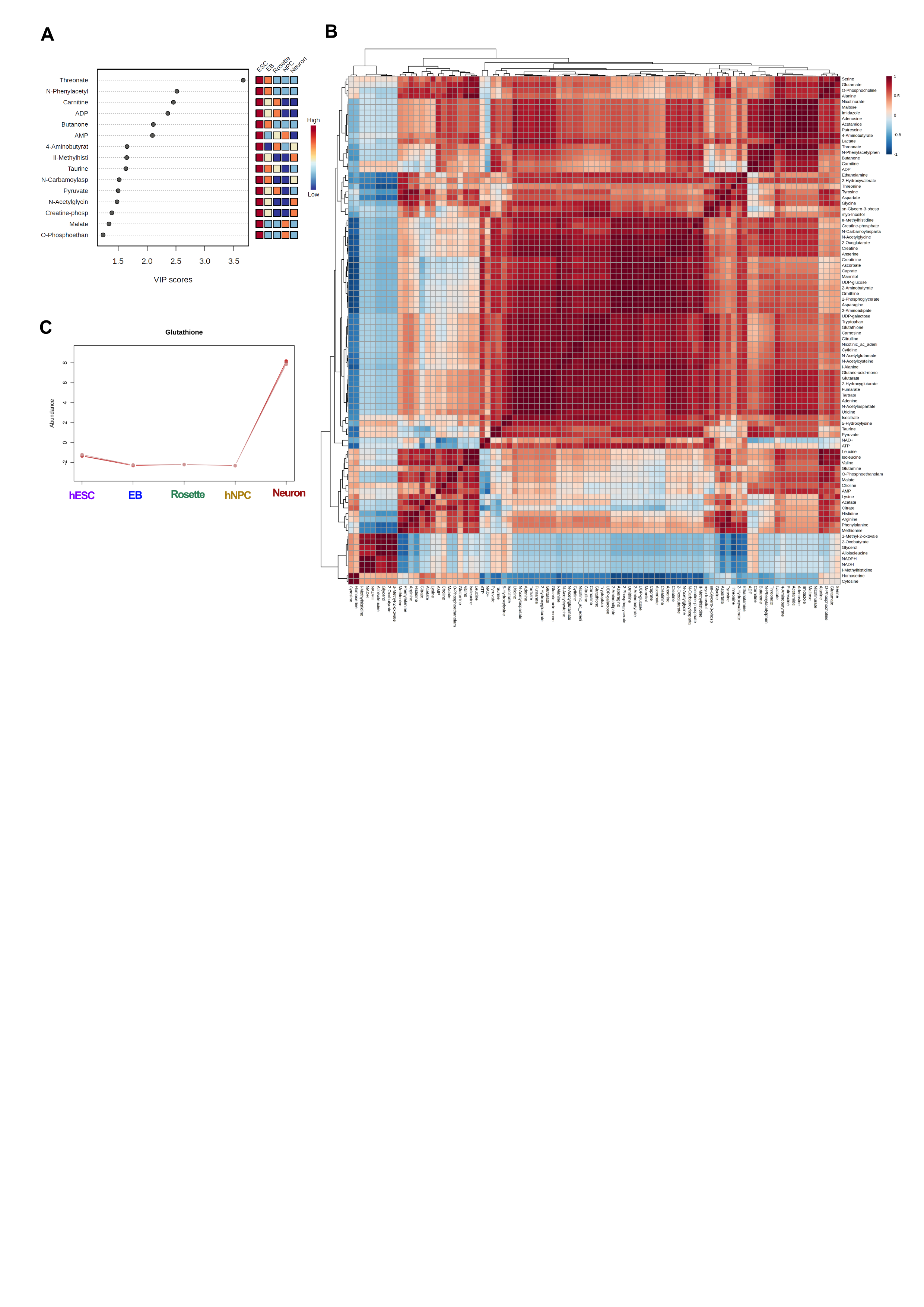

Supplement: SUPPLEMENTARY FIGURE S2 — (A) Important features identified by PLS-DA. The colored boxes on the right indicate the relative concentration of the corresponding metabolite in each group. (B) The overall correlation heatmap shows high correlation among purine-related metabolites (especially bioenergetic molecules) and nicotinamide-pathway related metabolites. (C) Specific distribution of glutathione across different cell-population shows higher abundance in neurons vs the rest of the samples. [file Image_2.tiff]

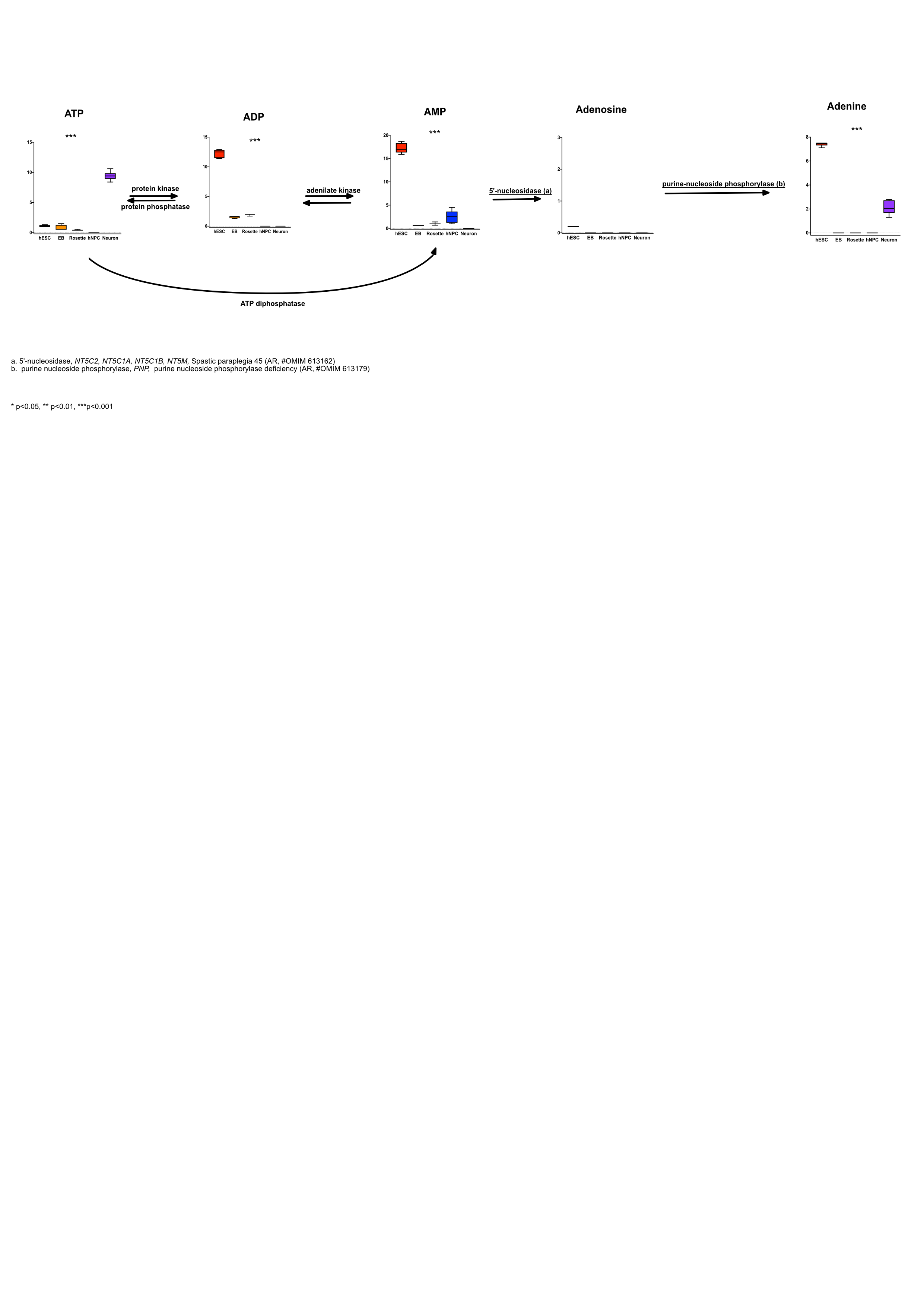

Supplement: SUPPLEMENTARY FIGURE S3 — ATP levels are significantly higher in neurons supporting the high energy rate (and OXPHOS-dependence) in these cells. [file Image_3.tiff]

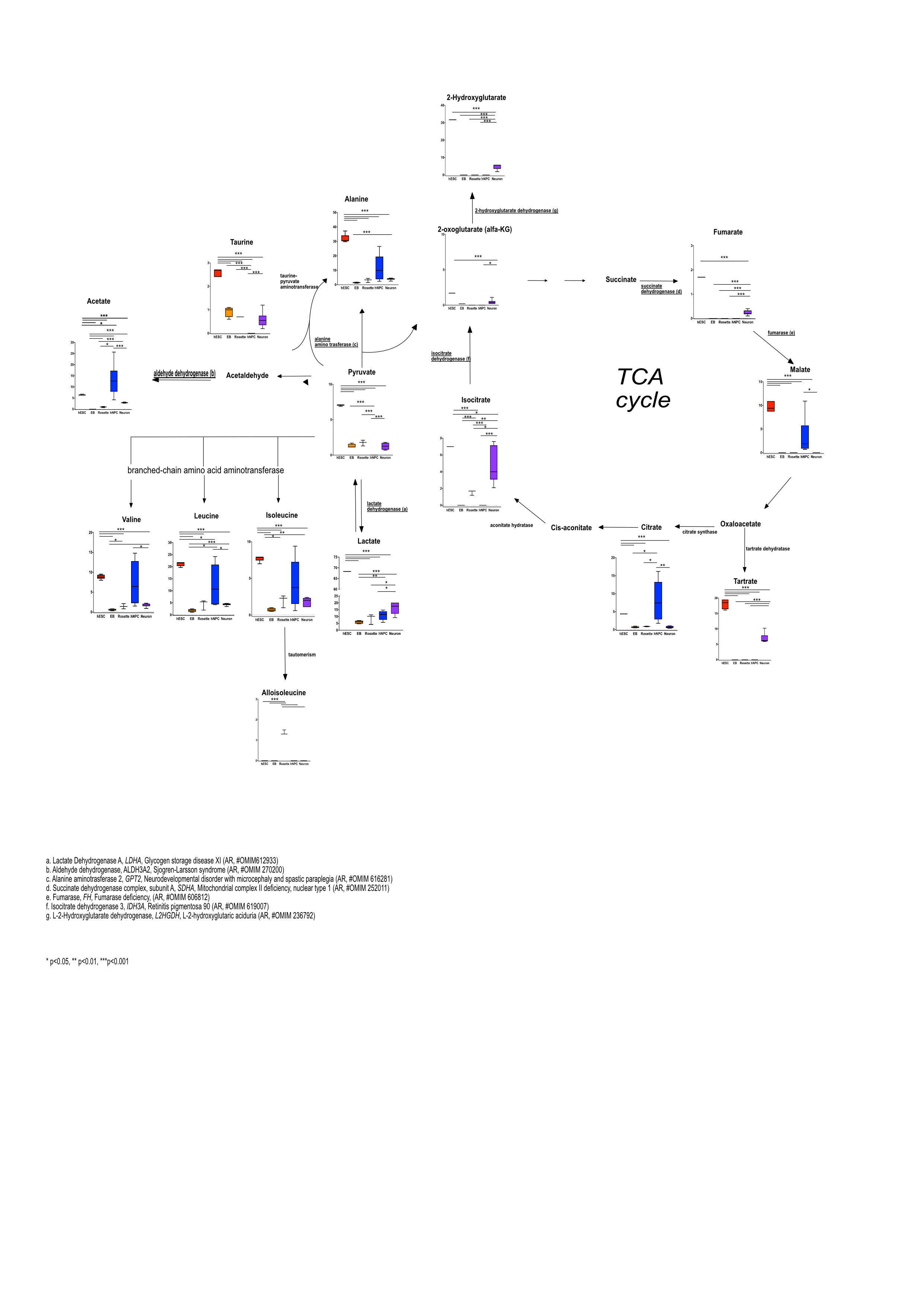

Supplement: SUPPLEMENTARY FIGURE S4 — TCA-related metabolites are more abundant in hESCs and neurons vs the other clusters, potentially related to their high energetic demands and substrates. Also, hESCs show the highest level of lactate and pyruvate, proving the high glycolytic state for these cells. [file Image_4.tiff]

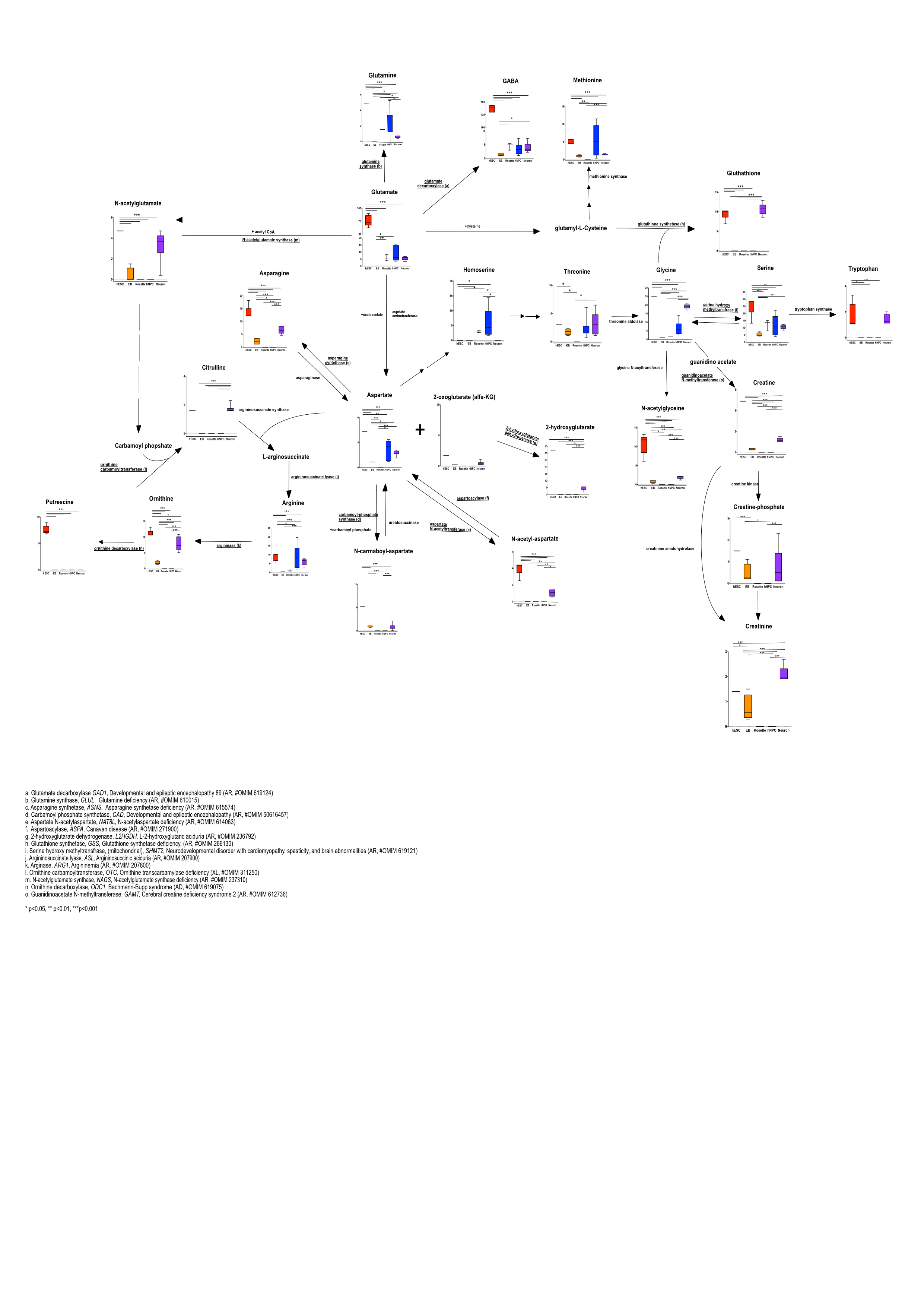

Supplement: SUPPLEMENTARY FIGURE S5 — Glutamate-related metabolites are shown for all the different groups (hESCs, EBs, rosettes, hNPCs, and neurons). In particular, high levels of glutamate and GABA were identified in hESCs vs the other clusters, while glutathione and creatinine levels were more abundant in neurons. [file Image_5.tiff]

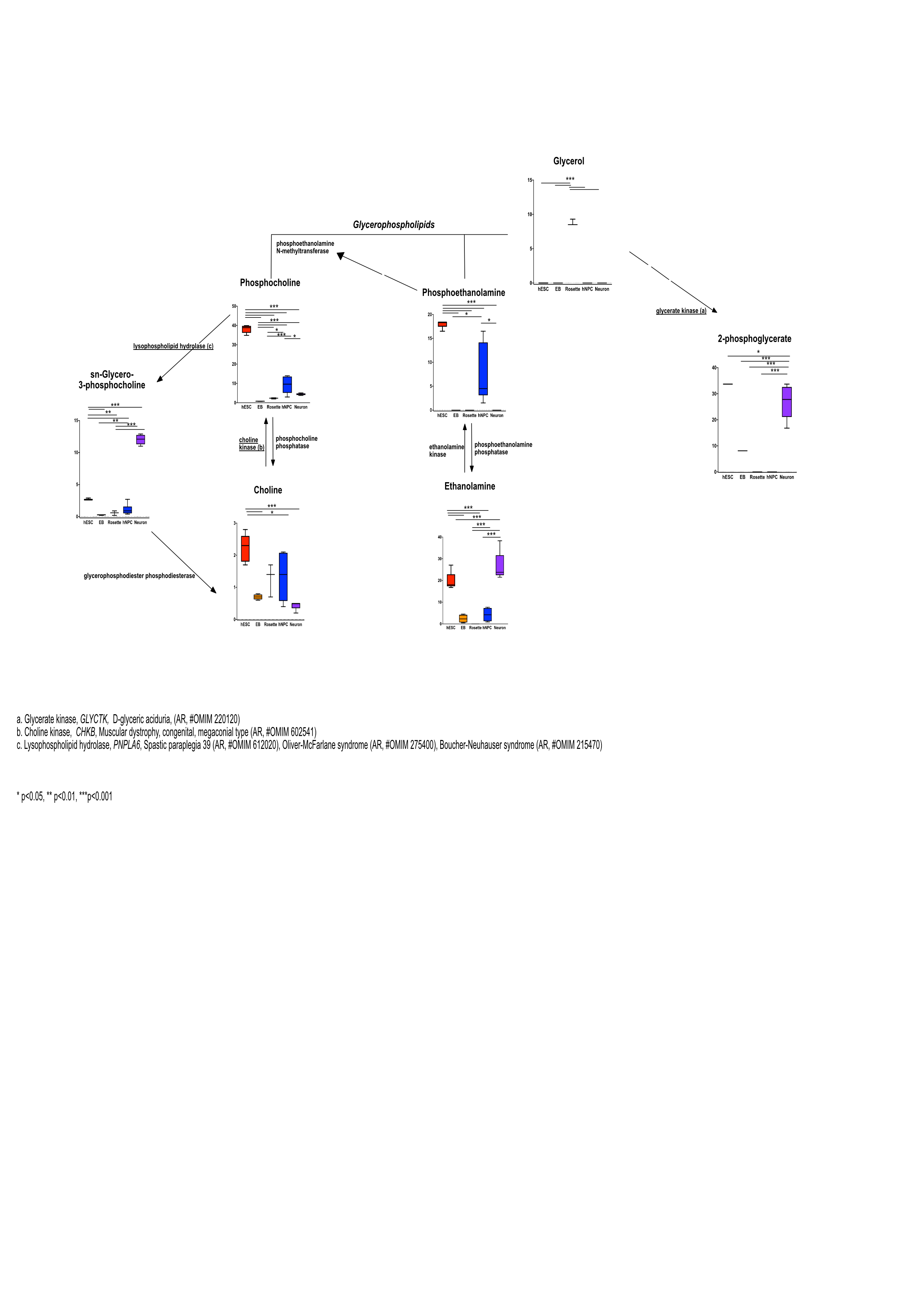

Supplement: SUPPLEMENTARY FIGURE S6 — High levels of glycerophophocholine were identified in neurons, while intermediate substrates like phosphoethanolamine and phosphocholine are more abundant in early precursors. [file Image_6.tiff]
